# Polylactic acid electrospun membrane loaded with cerium nitrogen co-doped titanium dioxide for visible light-triggered antibacterial photocatalytic therapy

**DOI:** 10.3389/fmicb.2024.1375956

**Published:** 2024-04-22

**Authors:** Hanlin Lv, Xiaomin Xia, Sa Sun, Zhaojun Niu, Jie Liu, Xue Li

**Affiliations:** ^1^Department of Stomatology, The Affiliated Hospital of Qingdao University, Qingdao, China; ^2^School of Stomatology, Qingdao University, Qingdao, China

**Keywords:** photocatalytic therapy, antibacterial, electrospinning, Ce-N co-doped, polylactic acid

## Abstract

Wound infection caused by multidrug-resistant bacteria poses a serious threat to antibiotic therapy. Therefore, it is of vital importance to find new methods and modes for antibacterial therapy. The cerium nitrogen co-doped titanium dioxide nanoparticles (N-TiO_2_, 0.05Ce-N-TiO_2_, 0.1Ce-N-TiO_2_, and 0.2Ce-N-TiO_2_) were synthesized using the hydrothermal method in this study. Subsequently, electrospinning was employed to fabricate polylactic acid (PLA) electrospun membranes loaded with the above-mentioned nanoparticles (PLA-N, PLA-0.05, PLA-0.1, and PLA-0.2). The results indicated that cerium and nitrogen co-doping tetrabutyl titanate enhanced the visible light photocatalytic efficiency of TiO_2_ nanoparticles and enabled the conversion of ultraviolet light into harmless visible light. The photocatalytic reaction under visible light irradiation induced the generation of ROS, which could effectively inhibit the bacterial growth. The antibacterial assay showed that it was effective in eliminating *S. aureus* and *E. coli* and the survival rates of two types of bacteria under 30 min of irradiation were significantly below 20% in the PLA-0.2 experimental group. Moreover, the bactericidal membranes also have excellent biocompatibility performance. This bio-friendly and biodegradable membrane may be applied to skin trauma and infection in future to curb drug-resistant bacteria and provide more alternative options for antimicrobial therapy.

## 1 Introduction

The drying up of the antibiotic discovery pipeline and the resulting spread of resistant pathogens have brought us to an antimicrobial resistance crisis (Lewis, 2020). At present, it is still difficult to predict the iteration of bacterial resistance, and the abuse of antibiotics in the medical environment has aggravated the emergence of bacterial resistance in environments such as water bodies (Huemer et al., 2020; Larsson and Flach, 2022). Therefore, the importance of finding a superior antimicrobial treatment method has been repeatedly emphasized. Recently, heavy ions, photothermal therapy (PTT), photodynamic therapy (PDT), and other antimicrobial therapy modes have been frequently reported (Gnanasekar et al., 2022). Among them, the photocatalytic antibacterial therapy based on titanium dioxide (TiO_2_) as photosensitizer (PS) has many advantages including superior biocompatibility, low cost, and controllable treatment procedure and does not induce bacterial resistance (Maleki et al., 2021).

Antimicrobial photocatalytic therapy generates reactive oxygen species (ROS) to eradicate bacteria by disrupting their structure and interfering with their normal physiological functions (Parra-Ortiz and Malmsten, 2022). However, both semiconductor materials and certain commercial PS also exhibit defects induced by the excitation source of ultraviolet light (UV). To address the limitations associated with UV, such as poor soft-tissue penetration due to its short wavelength and potential risk of skin cancer, it is imperative to transition toward therapeutic light sources that utilize visible or near-infrared light (Waiskopf et al., 2018).

TiO_2_ nanoparticles (TiO_2_ NPs) exhibit a potent antimicrobial effect against various pathogenic microorganisms. Silva et al. have investigated the efficacy of TiO_2_ NPs in eradicating pathogenic microorganisms such as *Escherichia coli (E. coli)*, *Staphylococcus aureus (S. aureus)*, and *Pseudomonas aeruginosa (P. aeruginosa)*, under 20 min of UV light excitation. The results manifested that the elimination efficiency of pathogenic microorganisms in aqueous media approached 100% (Rodrigues-Silva et al., 2017). Clemente et al. reported that after 2-h exposure to UV light, a 5-log reduction of *S. aureus* on the TiO2-coated surface was observed (Clemente et al., 2019). In a PBS solution of TiO_2_ NPs at a concentration of 500 mg/L, after 120 min of UV irradiation, *E. coli* cells decreased by 4.7-log (Chen et al., 2022). However, it should be noted that UV light exposure can potentially harm human skin tissues and its effectiveness against deep infections is limited due to the short wavelength (Gurusamy et al., 2019; Singh et al., 2019).

In order to narrow the band gap and enhance the photoconversion efficiency of TiO_2_ NPs in the visible light spectrum, common strategies involve doping TiO_2_ NPs with non-metallic elements such as nitrogen and carbon (Rengifo-Herrera et al., 2022). Nitrogen-doped TiO_2_ NPs exhibit a shifted band gap toward the visible spectrum. The composite resin loaded with nitrogen-doped TiO_2_ NPs demonstrated antibacterial effects against *Streptococcus mutans* (*S. mutans*) under visible light conditions (Ahmad Fauzi et al., 2022). Lee et al. have designed nitrogen-doped three-dimensional polycrystalline anatase TiO_2_ photocatalysts (N-3D TiO_2_) and killed more than 91.3% of *E. coli* strains after 10 cycles of visible light irradiation (Lee et al., 2013). Solís et al. reported that the presence of cerium (Ce) in Ce-doped TiO_2_ significantly redshifted its UV–visible absorption spectrum and reduced the band gap values of TiO_2_ (Solís et al., 2023). The addition of cerium as a dopant could result in the reduction in the size of TiO_2_ nanoparticles and enhance their photocatalytic activity. Meanwhile, cerium nanoparticles exhibit anti-inflammatory properties and effectively mitigate the autoimmune response (Nosrati et al., 2023). Moreover, the enhanced photocatalytic and antibacterial activities make them particularly promising for anti-viral applications (Naidi et al., 2021; Zandi et al., 2022).

Polylactic acid (PLA) is well known for its biodegradability, non-toxicity, elasticity, and rigidity. It is also a high polymer approved by the FDA for use in medical and food packaging (Bian et al., 2021). PLA membranes loaded with antimicrobial, anti-inflammatory, and bone-guiding materials fabricated by electrospinning technology can be used to accelerate wound healing and promote tissue repair (de Albuquerque et al., 2021). Shu et al. have developed a polylactic acid/nano-hydroxyapatite/Cu@ZIF-8 membrane to enhance the osteoconductive and antimicrobial properties of bone in the guided bone regeneration surgery (Shu et al., 2023). The graphene oxide-catechol hybrid/PLA membrane developed by Zhang et al. demonstrated favorable antibacterial properties against *S. aureus* and *E. coli* (Zhang et al., 2018). Zhao et al. synthesized silver(I) metal–organic framework-embedded PLA (Ag2(HBTC)/PLA), which could exhibit an inactivation efficiency of more than 99.9% against *E. coli* and *S. aureus* (Zhang et al., 2022). Antibacterial wound dressings based on PLA can effectively improve the microenvironment of the infected wound and maintain moisture in wounds, which has broad application prospects and application value.

**Figure 1:**
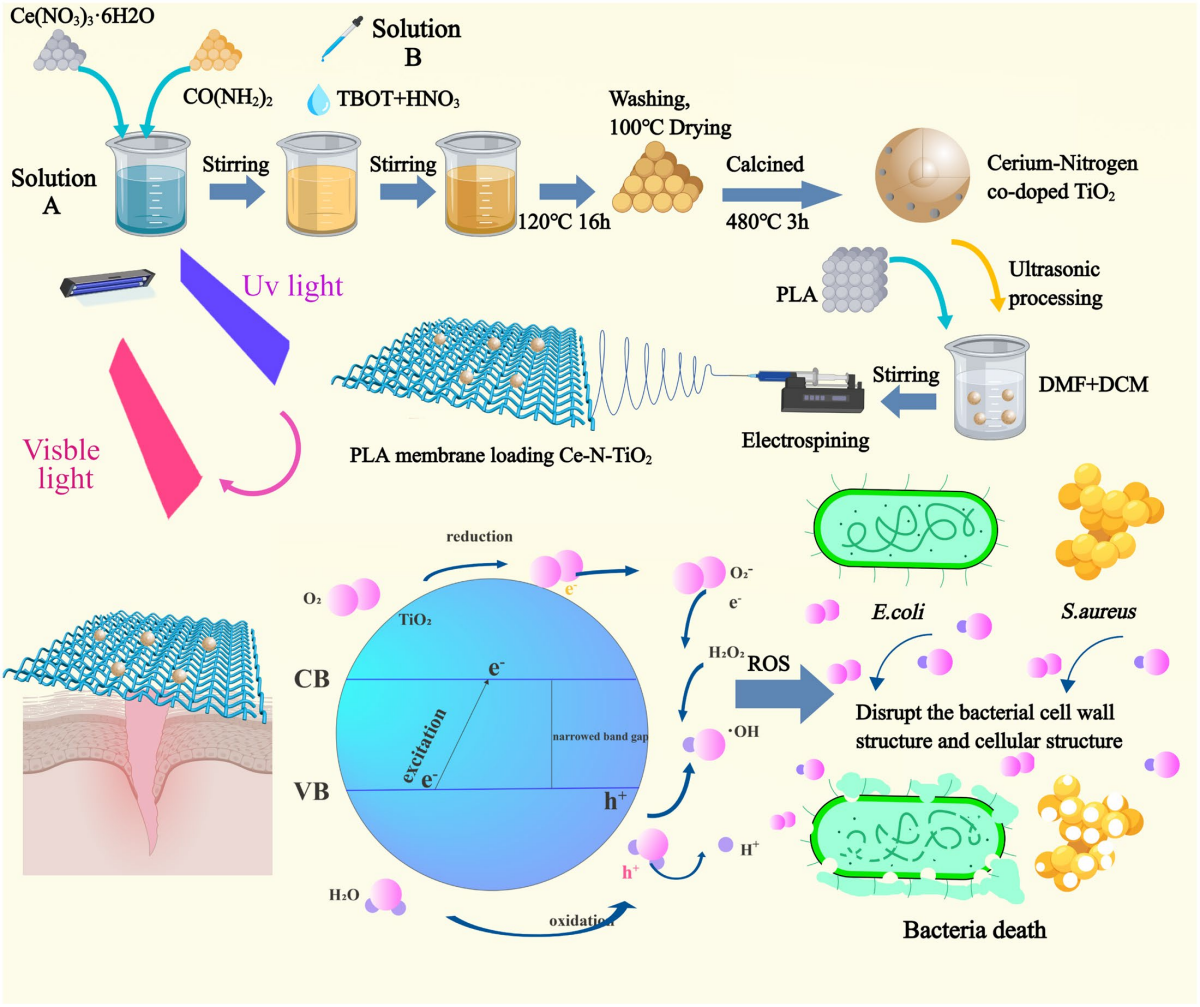
The preparation process of cerium nitrogen co-doped titanium dioxide nanoparticles and the fabrication of polylactic acid electrospinning membrane loaded with nanoparticles, along with an illustration depicting the photocatalytic reaction under visible light excitation for generating ROS to effectively eradicate *E. coli* and *S. aureus*.

In this study (Figure 1), nitrogen-doped TiO_2_ (N-TiO_2_) and N-TiO_2_ with three different cerium doping (0.05Ce-N-TiO_2_, 0.1Ce-N-TiO_2_, and 0.2Ce-N-TiO_2_) were fabricated through simple hydrothermal strategy. Then, the corresponding electrospun PLA membranes loaded with N-TiO_2_ and Ce-N-TiO_2_ were prepared (PLA-N, PLA-0.05, PLA-0.1, and PLA-0.2). Subsequently, the characterization of N-TiO_2_, Ce-N-TiO_2_, and PLA membranes was performed through X-ray diffraction (XRD), scanning electron microscope (SEM), transmission electron microscope (TEM), X-ray photoelectron spectroscopy (XPS), and Fourier transform infrared reflection (FTIR). Ultraviolet–visible diffuse reflectance spectra (UV–Vis-DRS) were used to calculate the band gap width. 1,3-Diphenylisobenzofuran (DPBF) was used to detect generated ROS through photocatalysis. 2,7-Dichlorodihydrofluorescein diacetate (DCFH-DA) was used to detect intracellular ROS. The *in vitro* antibacterial experiments demonstrated that PLA-0.1 and PLA-0.2 exhibited strong bactericidal activity against both *S. aureus* and *E. coli.* The biocompatibility of PLA membranes loaded with Ce-N-TiO_2_ was tested by CCK8 assay on L929 cells, showing excellent biocompatibility. Therefore, the as-mentioned PLA membrane loaded with Ce-N-TiO_2_ has a broad prospect in clinical application as a biodegradable antibacterial dressing for the treatment of infections.

## 2 Materials and methods

### 2.1 Materials

Glacial acetic acid (CH₃COOH), tetrabutyl titanate (TBOT), absolute ethyl alcohol (C_2_H_5_OH), urea (CO(NH_2_)_2_), nitric acid (HNO_3_), deionized water (DI), dichloromethane (DCM), dimethyl formamide (DMF) and cerium (III) nitrate hexahydrate (Ce(NO_3_)_3_·6H_2_O), and diphenylisobenzofuran (DPBF) were purchased from Aladdin Shanghai. Polylactic acid granules (4032D) were purchased from NatureWorks (USA). Luria–Bertani (LB) and Tryptic Soy Broth (TSB) culture media, agar powder, were procured from Haibo-bio (China).

### 2.2 Fabrication of Ce-N-TiO_2_ nanoparticles

Nitrogen-doped titanium dioxide (N-TiO_2_) and a series of cerium nitrogen co-doped TiO_2_ (Ce-N-TiO_2_) were prepared according to the methods of Meng et al. (2014) and Nasir et al. (2014). In total, 30 mL of ethanol and 2 mL of ultrapure water were taken into a 250-mL round-bottom flask fitted with a mechanical stirrer; 2 g of urea was used as nitrogen precursor, and varied amounts (0.05, 0.1, 0.2 g) of cerium nitrate hexahydrate (Ce(NO_3_)_3_6H_2_O) were totally added into the above solution to form “solution A.” In total, 30 mL of ethanol and 3 mL of 5 mol/L nitric acid solutions were added into another flask; 10 mL of TBOT was slowly added to this solution with continues stirring for 0.5 h to form “solution B.” Finally, solution B was added slowly into solution A at the rate of 1 mL/min under moderate stirring. Stirring was continued for another 2 h and then transferred into a 100-mL Teflon-lined autoclave, which was kept in the oven at 120°C for 16 h. After the autoclave was cooled to room temperature, the precipitates at the bottom of the autoclave were washed with deionized water several times to remove the impurities. The obtained samples were kept in the oven at 100°C overnight for drying. In order to have better crystallization and to remove the solvent and other impurities, the samples were calcined at 480°C for 3 h.

The obtained photocatalysts were denoted as N-TiO_2_, 0.05Ce-N-TiO_2_, 0.1Ce-N-TiO_2_, and 0.2Ce-N-TiO_2_, respectively; 0.05, 0.1, and 0.2 represent the grams of cerium nitrate added.

### 2.3 Electrospinning

PLA solution (8 *wt%*) was prepared by the addition of PLA particles into 8:2 (m: m) DCM/DMF mixture solvents and magnetic stirring for 20 h until well combined. Then, 5 *wt%* TiO_2_ NPs were dispersed in a DCM/CMF solvent through sonication for 30 min. The applied working distance (distance between the needle tip and collector), positive voltage, and flow rate were 20 cm, 20 kV, and 1 mL/h, respectively. The electrospinning environment was controlled at 25°C and 35% humidity, and the collection time was 4 h. The corresponding electrospun PLA membranes were PLA electrospun without TiO_2_ (PLA) and PLA electrospun loaded TiO_2_ (PLA-N, PLA-0.05, PLA-0.1, PLA-0.2). The PLA was vacuum dried at 60°C for 6 h to remove residual solvent.

### 2.4 Characterizations

The sample morphologies were characterized using a scanning electron microscope (SEM, JSM-6390LV, Japan) and a transmission electron microscope (TEM, JEM-2100Plus, Japan). Dynamic light scattering (DLS) measurements were performed by Zetasizer Nano ZS (Malvern, UK) equipped with a He-Ne laser (633 nm, 4 mW) with deionized water as the dispersant. The crystal structure of TiO_2_ was identified using an X-ray diffractometer (XRD, Smart Lab, Japan) with Cu-kα radiation (λ = 1.5406 Å). X-ray photoelectron spectroscopy (XPS) analysis was utilized to determine the surface chemical species of the particles (Escalab Xi+, Thermo Fisher, Czech Republic). The UV–visible diffuse reflectance spectra in the range of 200-800 nm were measured using a spectral electrochemical instrument (Autolab, Switzerland), with BaSO_4_ serving as the reflectance standard. The chemical bonds and other characteristics of the samples were analyzed using Fourier transform infrared spectroscopy (Nicolet iS50, Thermo Fisher, USA) to provide a comprehensive understanding of their properties.

### 2.5 The detection of reactive oxygen species (ROS)

The 1,3-diphenylisobenzofuran (DPBF) exhibits high specificity toward singlet oxygen (^1^O_2_), and after binding with singlet oxygen (^1^O_2_), DPBF will be irreversibly oxidized, and the absorption intensity at 410 nm will rapidly decrease. This enables the detection of ROS generation; 5.0 mL solution of acetonitrile containing DPBF (10 μg mL^−1^) was mixed with PLA, PLA-N, PLA-0.05, PLA-0.1, and PLA-0.2 electrospun samples (1.5 × 1.5 cm^2^). The DPBF solution (mixed with PLA electrospun) was then placed in a camera bellows. The PLA electrospun was irradiated with visible light from a 2mW cm^−2^ xenon lamp equipped with a UV filter for 30 minutes. The UV–Vis spectrophotometer (UV-2700, Japan) was utilized to measure the absorbance values of each solution at 415 nm during different time intervals.

The ROS Assay Kit (Beyotime, Shanghai) was used to measure the ROS in L929 cells treated with PLA, PLA-N, PLA-0.05, PLA-0.1, and PLA-0.2 electrospun samples. In short, L929 cells were seeded into six-well plates at 4 × 10^4^ cells per well and divided into seven groups: the first group was the blank control group; the second group was the positive control using Rosup reagent in the ROS Assay Kit. The medium containing nanoparticles was then replaced with serum-free medium supplemented with 10 uM DCFH-DA, and incubated at 37°C for 30 min. The cells were washed three times with serum-free medium and placed under an inverted fluorescence microscope for observation.

### 2.6 The detection of contact angle

The contact angle of water was characterized by using a contact angle analyzer (Krüss DSA30S, Germany). Distilled water (2 μL) was deposited onto each test surface to measure the contact angle.

### 2.7 The detection of swelling ratio

Briefly, the PLA-N, PLA-0.05, PLA-0.1, and PLA-0.2 membranes were initially weighed (dry weight) before immersion in PBS (pH 7.4) at 37°C for 2 and 24 h. The weights were specified as m_0_ before immersion and m_1_ after immersion. Therefore, the swelling ratio formula is swelling ratio = (m_1_–m_0_)/m_0_ × 100%.

### 2.8 Antibacterial experiments

*Staphylococcus aureus* (*S. aureus*, ATCC 6538) and *Escherichia coli* (*E. coli*, ATCC 8739) were purchased from Solarbio (Beijing) and selected as representative strains of Gram-positive (G+) and Gram-negative (G-) bacteria. Colony-forming unit (CFU) assay was used to assess the antibacterial properties of several PLA materials against the two bacteria. After being cultured for 24 h on the Tryptic Soy Broth (TSB) agar, the *S. aureus* colony was isolated and inoculated into a TSB fluid medium and shaking in a bacterial shaker speed of 150 rmp rotation at 37°C for 18 h. Similarly, after being cultured for 24 h on Luria–Bertani (LB) agar, the *E. coli* colony was carefully selected and inoculated into Luria–Bertani medium and shaken in a shaker at a rotation speed of 150 rpm rotation at 37°C for 18 h. Then, these two different bacterial suspensions were subsequently diluted to 10^6^ CFU/mL; 100 μL of the prepared bacterial suspension and PLA, PLA-N, PLA-0.05, PLA-0.1, and PLA-0.2 membranes (0.6 × 0.6 cm) were mixed in a 96-well plate. The antibacterial experiments were carried out under two conditions: one was a visible light source (2 mW cm^−2^ xenon lamp with a UV filter) irradiation for 30 min, which we called “Light”; and the other was without irradiation, which we called “Dark,” so as to compare and analyze the influence of visible light excitation Ce-N-TiO_2_ on bactericidal effect. After 30 min of dark treatment and visible light irradiation conditions, 50 μL of the bacterial suspension was taken out and diluted to 2 mL before being cultured in a shaker for 2 h. Then, the bacterial suspensions were, respectively, inoculated on TSB or LB agar and incubated at 37°C for 24 h. PLA was tested as a control (Wang et al., 2022). Colony-forming units (CFU) are counted, and the antibacterial efficiency of each plate is calculated as follows:


Antibacterialefficiency=A/B×100%

where *A* = CFU in the control group or CFU in the experiment group, and *B* = counts of colonies in the control group.

In order to observe the characteristics of bacteria growth and antibacterial effect more intuitively, bacteria growth curve tests were introduced into experiments. After undergoing the same experimental treatment, the bacterial suspension was divided into groups and cultured in individual wells of a 96-well plate at 37°C. PLA was also tested as a control. The absorbance change was measured every 2 h for 12 h using a microplate reader set to detect at 630 nm.

### 2.9 The detection of cytotoxicity assay

In this experiment, the cytotoxicity of PLA-N, PLA-0.05, PLA-0.1, and PLA-0.2 membranes was assessed through the CCK8 assay and calcein AM/PI live/dead kit (Beyotime, China). The L929 murine fibroblast cell culture medium was composed of Dulbecco’s Modified Eagle Medium (DMEM) with 10% fetal bovine serum (FBS) and 1% penicillin–streptomycin solution. In brief, the L929 cells cultured in flasks were digested using 0.25% trypsin–EDTA solution, resuspended in the medium for cell counting, and then, 200 μL cell suspension was seeded at a density of 1 × 10^4^ cells per well in 96-well plates incubated at 37°C with 5% CO_2_ for 24 h. Then, the 0.6 × 0.6 cm^2^ PLA electrospun membrane was carefully positioned onto the base of a 96-well plate. An experimental group was placed in a dark incubator, while another experimental group was exposed to light for 30 min before being placed in a dark incubator. The control group experiments without PLA membranes were executed in the same procedure as described before. After 18 h of incubation, the original culture medium in each well was removed, and 200 μL of fresh culture medium and 20 μL of CCK8 solution were added and incubated for an additional 2 h. The OD value at 450 nm was measured by a microplate reader (Molecular Devices, LLC). The cytotoxicity of the PLA membrane was calculated from the formula:


$$\text{Cell}\;\text{viability}=\left[\left(As-\text{Ab}\right)/\left(Ac-\text{Ab}\right)\right]\times 100\%$$

where *As* = experimental wells, *Ab* = blank wells, *and Ac* = control wells.

As the cell culture procedure described previously, the medium was removed and washed twice with PBS, and 100 μL calcein AM/PI was added to each well before incubation for 30 min in the dark. After incubation, the staining effect was observed under a fluorescence microscope (calcein AM was green fluorescence, Ex/Em = 494/517 nm; PI is red fluorescence, Ex/Em = 535/617 nm). Green fluorescence represents living cells and red fluorescence for dead cells.

### 2.10 Statistical analysis

The experiment was repeated at least three times. Experimental data were presented as mean ± standard deviation (SD) (*n* ≥ 3). Significant differences between data were tested by analysis of variance (ANOVA). In all evaluations, *p* < 0.05 was considered as statistically significant; *p* < 0.05, *p* < 0.01, and *p* < 0.001 were denoted by (*), (**), and (***), respectively.

## 3 Results and discussion

### 3.1 Characterization results

#### 3.1.1 XRD analysis

**Figure 2:**
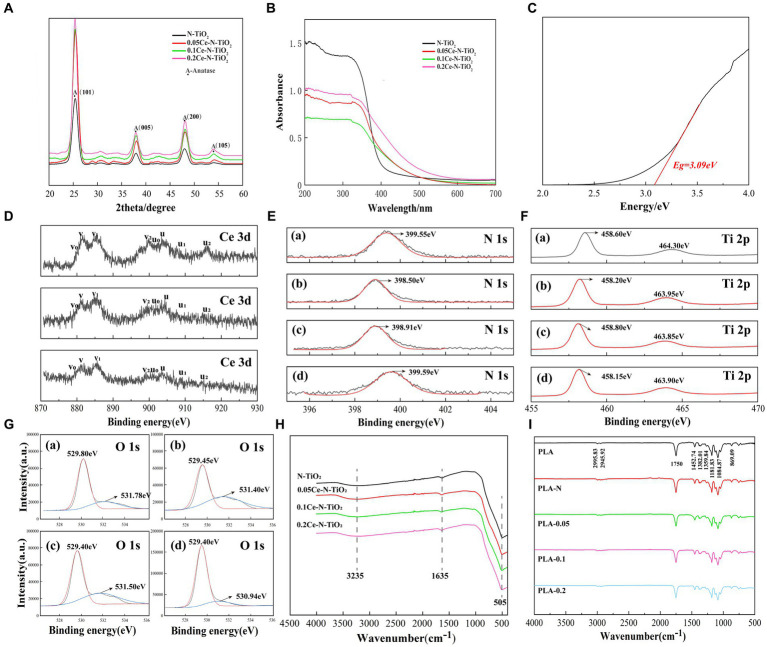
**(A)** XRD patterns of N-TiO_2_, 0.05Ce-N-TiO_2_, 0.1Ce-N-TiO_2_, and 0.2Ce-N-TiO_2_.NPs. **(B)** UV–visible diffuse reflectance spectra of N-TiO_2_, 0.05Ce-N-TiO_2_, 0.1Ce-N-TiO_2_, and 0.2Ce-N-TiO_2_.NPs. **(C)** The corresponding band gap of 0.1Ce-N-TiO_2_. XPS spectra analysis: **(D)** Ce 3d, **(E)** N 1s, **(F)** Ti 2p, **(G)** O 1s. **(A)** N-TiO_2_. **(B)** 0.05Ce-N-TiO_2_. **(C)** 0.1Ce-N-TiO_2_. **(D)** 0.2Ce-N-TiO_2_. **(H)** FTIR spectra of N-TiO_2_, 0.05Ce-N-TiO_2_, 0.1Ce-N-TiO_2_, and 0.2Ce-N-TiO_2_. **(I)** FTIR spectra of PLA, PLA-N, PLA-0.05, PLA-0.1, and PLA-0.2.

As shown in Figure 2A, after analyzing the XRD results of N-TiO_2_, 0.05Ce-N-TiO_2_, 0.1Ce-N-TiO_2_, and 0.2Ce-N-TiO_2_, the emission peaks of samples (101, 005, 200, and 105) were consistent with the XRD characteristic peaks of anatase TiO_2_. Pure TiO_2_ samples typically exhibited XRD emission characteristics of both anatase and rutile phases (Leite et al., 2020). Therefore, the introduction of N-element doping and Ce-N elements co-doping was conducive to the formation of the anatase phase in TiO_2_. Anatase TiO_2_ exhibited superior photocatalytic activity than the rutile phase due to the structural differences caused by band gap width and conduction band potential (Gopinath et al., 2020). It should be noted that no peaks related to dopants were detected in the XRD images, and it may be due to its low content. The other case was the incorporation of dopants into the TiO_2_ constructure, replacing titanium or oxygen atoms in the lattice or occupying interstitial positions at the interstitial site.

#### 3.1.2 UV–visible diffuse reflectance spectra analysis

The UV–visible diffuse reflectance spectra of N-TiO_2_, 0.05Ce-N-TiO_2_, 0.1Ce-N-TiO_2_, and 0.2Ce-N-TiO_2_ prepared as described above are shown in Figure 2B. The absorption intensity of cerium nitrogen co-doped TiO_2_ NPs exhibited a significant enhancement in the visible light range, and 0.2Ce-N-TiO_2_ demonstrated the most obvious improvement. Furthermore, compared to N-TiO_2_, the optical absorption edge of Ce-N-TiO_2_ samples was remarkably redshifted to the visible region. The redshift in absorption can be attributed to the transition between Ce^4+^ and Ce^3+^, where the ground-state electrons of Ce^3+^ are excited to the Ce 4f level (Bian et al., 2021). Figure 2C illustrates the band gap width of determined 0.1 Ce-N-TiO_2_, which was reduced to a certain extent relative to the undoped TiO_2_ (≈3.2 eV) (Fouad et al., 2020). The band gap widths of the TiO_2_ NPs were 3.26 eV(N-TiO_2_), 3.18 eV (0.05Ce-N-TiO_2_), 3.09 eV (0.1Ce-N-TiO_2_), and 2.91 eV (0.2Ce-N-TiO_2_), respectively, indicating that Ce-N co-doping was effective in reducing the band gap width and enhancing the photocatalytic efficiency of TiO_2_ NPs under visible light conditions (Bian et al., 2021).

#### 3.1.3 XPS analysis

To characterize the surface elements and their chemical states of the prepared N-TiO_2_, 0.05Ce-N-TiO_2_, 0.1Ce-N-TiO_2_, and 0.2Ce-N-TiO_2_, the XPS images were recorded. Figure 2D depicts the binding region of Ce 3d, while the core-level spectra of Ce 3d exhibit relatively complex characteristics that are related to the final-state occupation of the Ce 4f level and hybridization of Ce 4f and O 2p (Song et al., 2008; Lu et al., 2019). The spin-orbitals coupling states of 3d_5/2_ and 3d_3/2_ are denoted by v and u, respectively. For instance, in Figure 2D, the binding energies of Ce 3d_5/2_ at 879.80, 881.50, 885.85, and 899.95 eV were labeled as v_0_, v, v_1_, and v_2_, the binding energies of Ce 3d_5/2_ at 879.80, 881.50, 885.85, and 899.95 eV were labeled as v_0_, v, v_1_, and v_2_, respectively, while the binding energies of Ce 3d_3/2_ at 902.35, 903.75, 909.00, and 915.05 eV were labeled as v_0_, v, v_1_, and v_2_. The emission peaks at v, v_1_, v_2_, u, and u_2_ were correlated to Ce^4+^. v_2_/u_2_ was associated with the Ce(3d^9^4f^0^) (O 2p^6^) final state and v/u with the primary light emission in the Ce(3d^9^4f^1^) (O 2p^5^) final state. Furthermore, the emission peaks observed at v_0_, u_0_, and u_1_ correspond to characteristic binding energies of Ce^3+^ configurations between the O 2p level and Ce 4f level. The peak of u_1_ can be attributed to the final state of Ce(3d^9^4f^2^) (O 2p^5^), while v_0_/u_0_ could be originated from the final state of Ce(3d^9^4f^1^) (O 2p^6^) (Zhao et al., 2014b; Mathew et al., 2023). Therefore, the above characterization demonstrates the co-existence of Ce^3+^ and Ce^4+^ in different valence states within Ce-N-TiO_2_ NPs. The photocatalytic efficiency of Ce-N-TiO_2_ can be enhanced by the co-existence of Ce^3+^ and Ce^4+^ on its surface, which effectively suppresses the recombination of photogenerated charge carriers (Morlando et al., 2020).

A single N 1 s emission peak was clearly observed in Figure 2E, with binding energies of 399.55, 398.50, 398.91, and 399.59 eV for N-TiO_2_, 0.05Ce-N-TiO_2_, 0.1Ce-N-TiO_2_, and 0.2Ce-N-TiO2, respectively. The results indicate that the doped nitrogen species occupied an interstitial position, forming a direct bond with lattice oxygen, and may exist in a state of Ti-O-N or Ti-N-O (Zhao et al., 2014a). As shown in Figure 2F, in the XPS spectra of the Ti 2p region, there were two distinct emission peaks of the Ti 2p region. Taking Figure 2F:b as an example, the emission peaks at 458.20 eV (Ti 2p_3/2_) and 463.95 eV (Ti 2p_1/2_) indicated that Ti mainly exists in the form of Ti^4+^ (Yu et al., 2016). The two Ti 2p binding energy peaks of Ce-N co-doped TiO_2_ exhibited a slight shift toward lower energy values in comparison with N-TiO_2_. This suggested that the presence of Ce dopant altered the chemical environment surrounding Ti^4+^ in the mixed oxide of Ce-Ti and may be potentially influencing the interaction between Ti and N species (Cheng et al., 2014). Figure 2G shows the peak-fitted XPS spectra of the O 1 s region of N-TiO_2_ and Ce-N-TiO_2_ NPs. It is evident that the O 1 s spectra of XPS exhibited two distinct oxidation chemical states, including lattice oxygen (O_L_) and chemo-adsorbed oxygen (O_H_). As shown in Figure 2G:a, the peak at 529.80 eV of O 1 s is attributed to the O_L_, and the peak at 531.78 eV was associated with surface hydroxyl groups that belong to the feature of chemo-adsorbed oxygen (Gomez et al., 2015). A comparison of the four O1s spectra in Figure 2G shows that the O_H_ content of Ce-N-TiO_2_ was significantly higher than that of N-TiO_2_. It also demonstrated an increasing trend with higher Ce-N co-doping ratios. Additionally, it has been reported that surface O_H_ exhibits higher mobility than lattice oxygen, which facilitates the trapping of photogenerated electrons and enhances the separation efficiency of charge carriers. Simultaneously, it effectively generates surface free radicals that play a crucial role in oxidation reactions and contribute to improving photocatalytic efficiency and photon utilization. Additionally, the significant shift of the Ti-O bond energy toward the lower binding energy region in the Ce-N co-doped TiO_2_ sample may be attributed to the substitution of the N-element for the lattice oxygen atoms in TiO_2_, thereby increasing its probability charge density, which is also consistent with the previous analysis of XPS N1s spectra (Stiehl et al., 2004; Zhao et al., 2014b; Monmaturapoj et al., 2018; Zhang et al., 2020; Gnanasekar et al., 2022).

#### 3.1.4 FTIR analysis

Figures 2H,I show the Fourier infrared spectra of N-TiO_2_, 0.05Ce-N-TiO_2_, 0.1Ce-N-TiO_2_, and 0.2Ce-N-TiO_2_ and PLA, PLA-N, PLA-0.05, PLA-0.1, and PLA-0.2. As shown in Figure 2H, three distinct characteristic peaks were observed at 505, 1635, and 3235 cm^−1^, respectively. Among them, the absorption peak at 3235 cm^−1^ was attributed to the O-H stretching vibration of adsorbed water on the material surface, while the absorption peak at 1635 cm^−1^ corresponded to the H-O-H bending vibration of water molecules. Additionally, the characteristic TiO_2_ absorption peak near 505 cm^−1^ was associated with Ti-O bond stretching vibrations (Chen et al., 2018; Monmaturapoj et al., 2018; Awasthi et al., 2021). As shown in Figure 2I, the double absorption peaks at 2995.83 cm^−1^ and 2945.92 cm^−1^ were due to the vibration of C-H groups stretching. The absorption peaks at 1750 cm^−1^ and 1452.74 cm^−1^ were associated with the presence of C=O and -C-CH_3_ groups, respectively; while peaks at 1181.83 and 1084.87 cm^−1^ were attributed to the C-O stretching of ester group, peaks at 1381.83 cm^−1^ and 1359.84 cm^−1^ were associated with the bending of -C-H and peak at 869.09 cm^−1^ represents extension of -C-C (Idris et al., 2015; Dong et al., 2021; Zheng et al., 2023). As shown in Figure 2I, the FTIR spectra of PLA loaded with different TiO_2_ NPs resembled to that of pure PLA electrospun membrane, and no additional emission peaks corresponding to chemical groups were observed, indicating the physical binding mode. In addition, the change in emission peak intensity of PLA membrane loaded with different TiO_2_ NPs was not particularly significant, which suggested an interaction between the TiO_2_ NPs and the PLA. The absence of Ti-O bond peaks may be attributed to the low concentration of TiO_2_ present (Li et al., 2020; Andrade-Guel et al., 2022).

#### 3.1.5 SEM, TEM analysis, and dynamic light scattering (DLS) measurements

**Figure 3:**
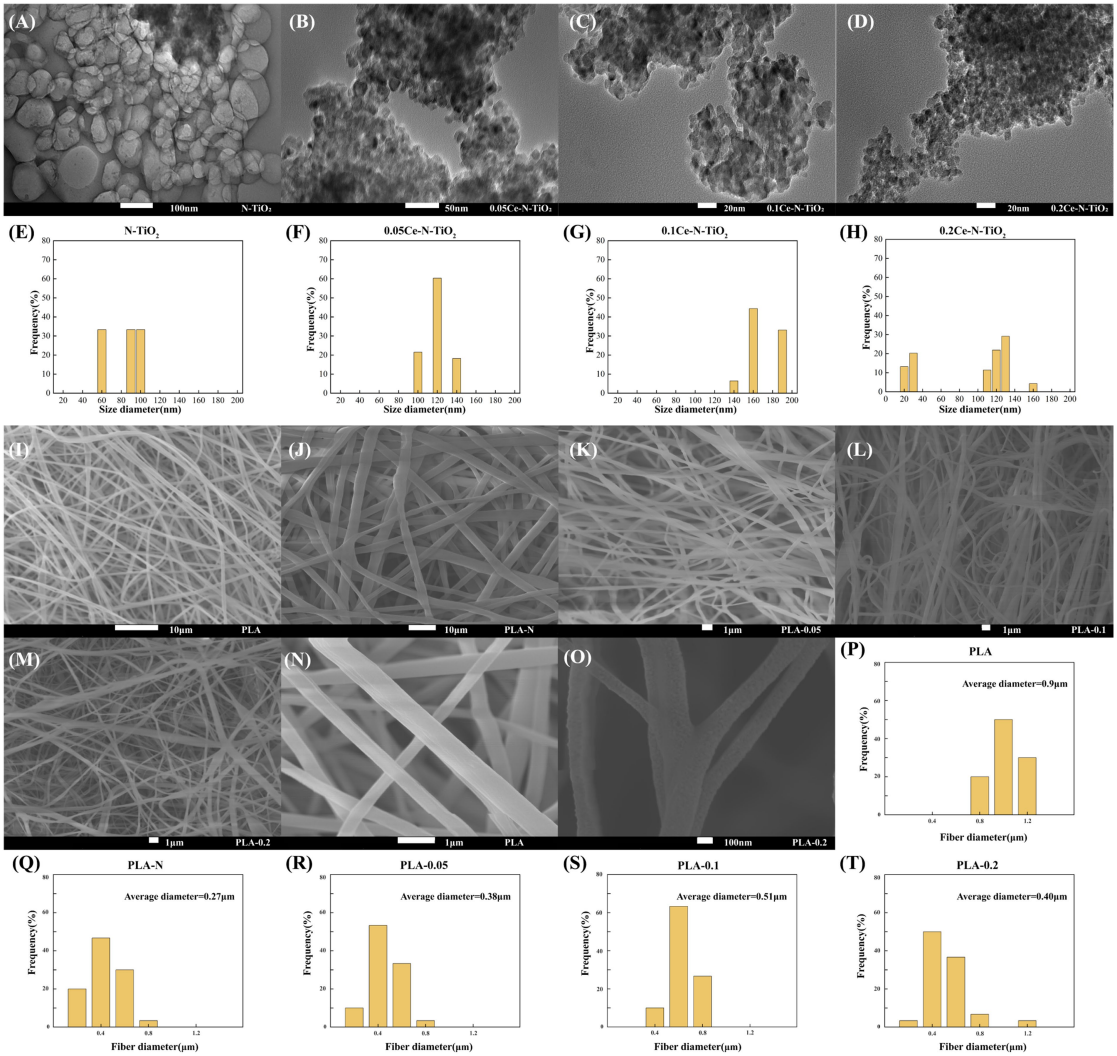
TEM images of **(A)** N-TiO_2_; **(B)** 0.05Ce-N-TiO_2_; **(C)** 0.1Ce-N-TiO_2_; and **(D)**. 0.2Ce-N-TiO_2_. DLS particle size distribution images: **(E)** N-TiO_2_, **(F)** 0.05Ce-N-TiO_2_, **(G)** 0.1Ce-N-TiO_2_, **(H)** 0.2Ce-N-TiO_2_. SEM micrographs of **(I)** PLA; **(J)** PLA-N; **(K)** PLA-0.05; **(L)** PLA-0.1; and **(M)** PLA-0.2. **(N)** The morphology of PLA electrospun fibers at high magnification. **(O)** The morphology of PLA-0.2 electrospun fibers at high magnification. Fiber diameter distributions of PLA electrospun: **(P)** PLA, **(Q)** PLA-N, **(R)** PLA-0.05, **(S)** PLA-0.1, and **(T)** PLA-0.2.

The TEM images of N-TiO_2_, 0.05Ce-N-TiO_2_, 0.1Ce-N-TiO_2_, and 0.2Ce-N-TiO_2_ are shown in Figures 3A–D. In Figure 3A, spherical or elliptical N-TiO_2_ NPs with varying size distributions and an average diameter of 81.76 nm could be observed. As illustrated in Figures 3B–D, nanoparticles were formed in large random blocks (approximately 80–300 nm), due to the particle agglomeration. With the increase in cerium nitrogen co-doping ratio, TiO_2_ NPs exhibited a more regular shape and smaller grain size. As shown in Figure 3D, the polyhedral particle structure, which was primarily composed of irregular hexagons and rhomboids could be clearly discernible in 0.2Ce-N-TiO_2_ and exhibiting a particle diameter ranging from 5 to 20 nm.

The particle size distribution of a dispersion system can be characterized using dynamic light scattering (DLS). This technique is based on the properties of multiple scattering and Brownian motion of nanoparticles and analyzes the scattered light generated when the nanoparticles are illuminated by a laser (Ramos et al., 2017). As shown in Figure 2E, the average particle size distribution of most N-TiO_2_ NPs was concentrated within the range of 90–100 nm and 60 nm. In contrast, as shown in Figures 2F–H, the average particle size distribution of Ce-N-TiO_2_ was larger than N-TiO_2_, and 0.2Ce-N-TiO_2_ was concentrated in the range of 100–140 nm, or 20 nm and 160 nm. At the same time, a considerable part of 0.2Ce-N-TiO_2_ particle size was distributed approximately 130 nm. The small size and large specific surface area of nanoparticles contribute to their higher surface energy, and the occurrence of agglomeration is prone to happen. This trend becomes more evident as the size of nanoparticles in the 1–100 nm range decreases (Starsich et al., 2019; Sazali et al., 2023). Although we could observe from the TEM results (Figures 3A–D) that the size of nanoparticles gradually decreases with the gradual increase of Ce doping amount, the agglomeration phenomenon became more and more obvious. Consequently, the particle size measured by DLS was significantly larger than the actual diameter of the Ce-N-TiO_2_ single particle. Therefore, the presence of aggregates resulted in the emergence of multi-distribution in Figures 2E,H. It also made the particle size distribution range of 0.05Ce-N-TiO_2_ concentrated in 100–140 nm, while for 0.1Ce-N-TiO_2_, it concentrated at 140 nm, 160 nm, 190 nm, and 220 nm (Samadi et al., 2018).

Figures 3I–O show the SEM image of the PLA, PLA-N, PLA-0.05, PLA-0.1, and PLA-0.2 electrospun; Figures 3N,O show the SEM micrographs of PLA electrospun fibers and PLA-0.2 electrospun, respectively. The pure PLA fibers exhibited a uniformity in shape and a relatively smooth surface texture. In Figures 3J–M, with the introduction of N-TiO_2_, 0.05Ce-N-TiO_2_, 0.1Ce-N-TiO_2_, and 0.2Ce-N-TiO_2_ into the solution system, the fiber morphology of PLA has changed. The uniformity of fiber diameter distribution was significantly reduced with the formation of bead-like structures in some of the electrospun fibers. Figures 3P–T illustrate the average fiber diameter and its distribution of these PLA electrospun, which were calculated based on SEM images. It is evident that the PLA-N, PLA-0.05, PLA-0.1, and PLA-0.2 nanocomposite exhibited a significantly reduced average fiber diameter compared to pure PLA, with a less concentrated distribution of fibers and more fibers distributed in the range of 0.4–0.5 μm, while pure PLA displayed a more concentrated fiber distribution in the range of 0.8–1.0 μm.

The morphology of electrospun fibers is influenced by various factors, including voltage, air humidity, temperature, collector distance, solvent system, and molecular weight (Xue et al., 2019). The rough structure and bead formation of fibers can be attributed to the high concentration of TiO_2_ NPs in the solvent that cannot be well dispersed. The rough structure and bead formation during electrospinning may be attributed to the high concentration of TiO_2_ NPs in the solvent, which cannot be homogeneously dispersed. Due to the volatility of the solvent, NPs tend to accumulate at the needle orifice, resulting in poor fluidity of the spinning solution and incomplete stretching of droplets. Moreover, the addition of nanoparticles to the PLA mixture resulted in a reduction in the diameter of electrospun nanofibers due to the induced charge density of TiO_2_ NPs in the solution. TiO_2_ NPs also elevate the viscosity of the PLA solution, resulting in irregular PLA fiber formation (Bhattacharjee, 2016; Zehlike et al., 2019).

### 3.2 Contact angle detection

**Figure 4:**
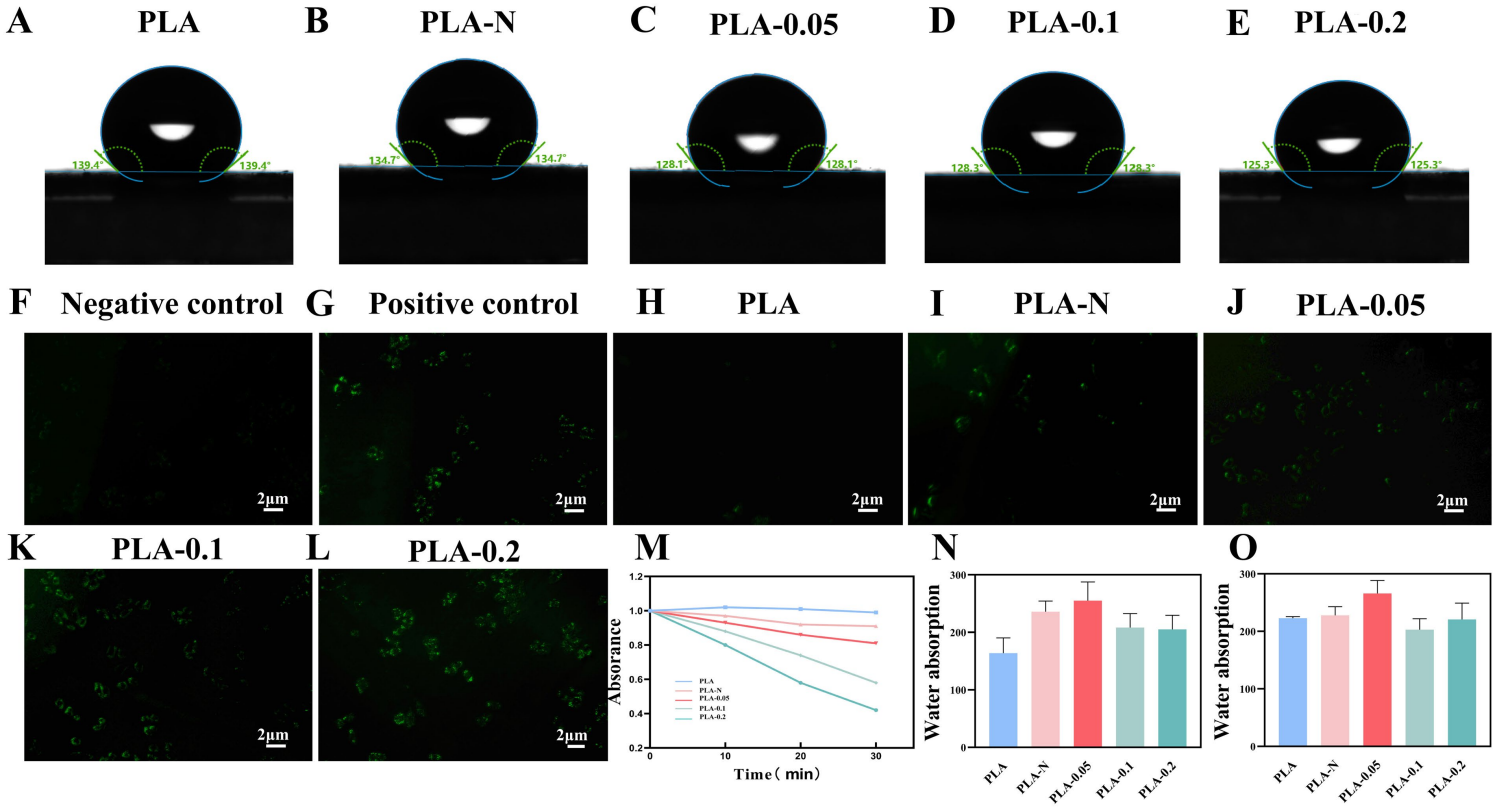
**(A–E)** Contact angle of PLA, PLA-N, PLA-0.05, PLA-0.1, and PLA-0.2 electrospinning membranes. Fluorescence microscope images of intracellular ROS generation induced by PLA, PLA-N, PLA-0.05, PLA-0.1, and PLA-0.2 with DCF-DA: **(F)** negative control, **(G)** positive control, **(H)** PLA, **(I)** PLA-N, **(J)** PLA-0.05, **(K)** PLA-0.1, and **(L)** PLA-0.2. **(M)** Absorption curves of DPBF solution after incubation with different PLA, PLA-N, PLA-0.05, PLA-0.1, and PLA-0.2. **(N)** Swelling ratio of PLA, PLA-N, PLA-0.05, PLA-0.1, and PLA-0.2 electrospinning membranes at 2 h. **(O)** Swelling ratio of PLA, PLA-N, PLA-0.05, PLA-0.1, and PLA-0.2 electrospinning membranes at 24 h.

The membrane surface hydrophobicity–hydrophilicity properties were investigated by water contact angle analysis. As shown in Figures 4A–E, most of the membranes exhibit hydrophobic properties with contact angles ranging from 140° to 125°. With the addition of TiO_2_ NPs, the contact angles of PLA-N, PLA-0.05, PLA-0.1, and PLA-0.2 films declined to a certain extent, indicating that the water wettability of PLA membranes was improved.

### 3.3 The detection of ROS

ROS plays a pivotal role in photodynamic antibacterial therapy (PDT), photocatalytic antibacterial therapy (PCT), and sonodynamic antibacterial therapy (SDT) modalities. Sufficient ROS, including superoxide anion radical (O_2_^·−^), hydrogen peroxide (H_2_O_2_), singlet oxygen (^1^O_2_), and hydroxyl radical (·OH), can impair bacterial membrane phospholipids and membrane proteins, leading to the disruption of bacterial structure integrity, intracellular substance leakage, functional inactivation of the membrane transport system, and related protease. Moreover, ROS can irreversibly damage the components of DNA, thereby causing double-strand breaks and inhibiting the growth and reproduction of bacteria (Jia et al., 2019; Feng et al., 2021).

The fluorescent probe DCFH-DA (2,7′-dichlorodihydrofluorescein diacetate) is widely employed for intracellular ROS testing due to its good lipophilicity and membrane permeability. Upon oxidation by ROS, DCFH yields 2′,7′-dichlorofluorescein (DCF), which exhibits intense fluorescence that can be visualized using fluorescence microscope.

Figures 4F–L show the images of intracellular ROS staining; Figures 4F,G show the negative control group and positive control group, respectively. Figures 4H–L exhibit a gradual increase in fluorescence intensity, indicating elevated intracellular ROS levels under visible light treatment with PLA-N, PLA-0.05, PLA-0.1, and PLA-0.2. Notably, PLA-0.2 displayed the highest fluorescence intensity, suggesting that PLA-0.2 enhanced intracellular ROS the most and was more conducive to playing a bactericidal role (Mickymaray et al., 2023).

1, 3-diphenylisobenzofuran (DPBF) is a highly specific fluorescent probe for singlet oxygen (^1^O_2_), which serves as an indicator of its presence. As shown in Figure 4M, after 30 min of exposure to light, the PLA group did not exhibit a significant decrease in absorbance at 411 nm. In the other four groups, the absorbance of DPBF solution at 411 nm exhibited a continuous decrease with increasing light exposure time, particularly in PLA-0.2. These results proved that the PLA membranes loaded with Ce-N-TiO_2_ increased their photocatalytic activity and produced more ROS under visible light conditions as the proportion of Ce doped with TiO_2_ increased.

### 3.4 Swelling ratio detection

For medical biological dressings, the evaluation system must include properties such as water absorption and swelling ratio, which are crucial for creating a favorable wound microenvironment and promoting healing (Hajikhani et al., 2021). The maintenance of wound cleanliness and infection control is essential throughout all stages of wound healing. Additionally, a moist wound that is not too dry or wet plays a crucial role in wound treatment in order to accelerate the healing process (Powers et al., 2016). In addition, the normal exudation of tissue fluid or blood from the wound, along with the inflammatory secretion from an infected wound, significantly impacts the microenvironment of the wound and ultimately influences the entire process of wound recovery. Therefore, the dressings used to cover the wound surface should possess excellent water absorption and moisturizing properties, in addition to antibacterial activity and good biocompatibility (Liang et al., 2021; Peng et al., 2022; Zeng et al., 2022).

The 2 h and 24-h swelling ratio performance is shown in Figures 4N,O. After immersion in PBS for 2 h, the water absorption of the pure PLA electrospun membranes prepared according to the parameters described above was close to 160%, while the rate of the PLA-N, PLA-0.05, PLA-0.1, and PLA-0.2 electrospinning membranes significantly increased to different degrees. In addition, after immersion in PBS for 24 h, the swelling ratio of PLA, PLA-N, PLA-0.05, PLA-0.1, and PLA-0.2 was approximately 220%. Among them, the ratio of PLA-0.05 was slightly higher, approximately 260%. It was plausible that the hydrophilic properties of TiO_2_ NPs within PLA may be accountable. This also could be attributed to TiO_2_, which can easily penetrate the unsaturated bonds surrounding the PLA polymer chain, thereby enhancing its capillary effect and increasing water absorption through this effect. Simultaneously, changes in diameter distribution and void ratio of the electrospun membrane may also improve the swelling ratio.

### 3.5 Antibacterial activity of PLA, PLA-N, PLA-0.05, PLA-0.1, PLA-0.2

**Figure 5:**
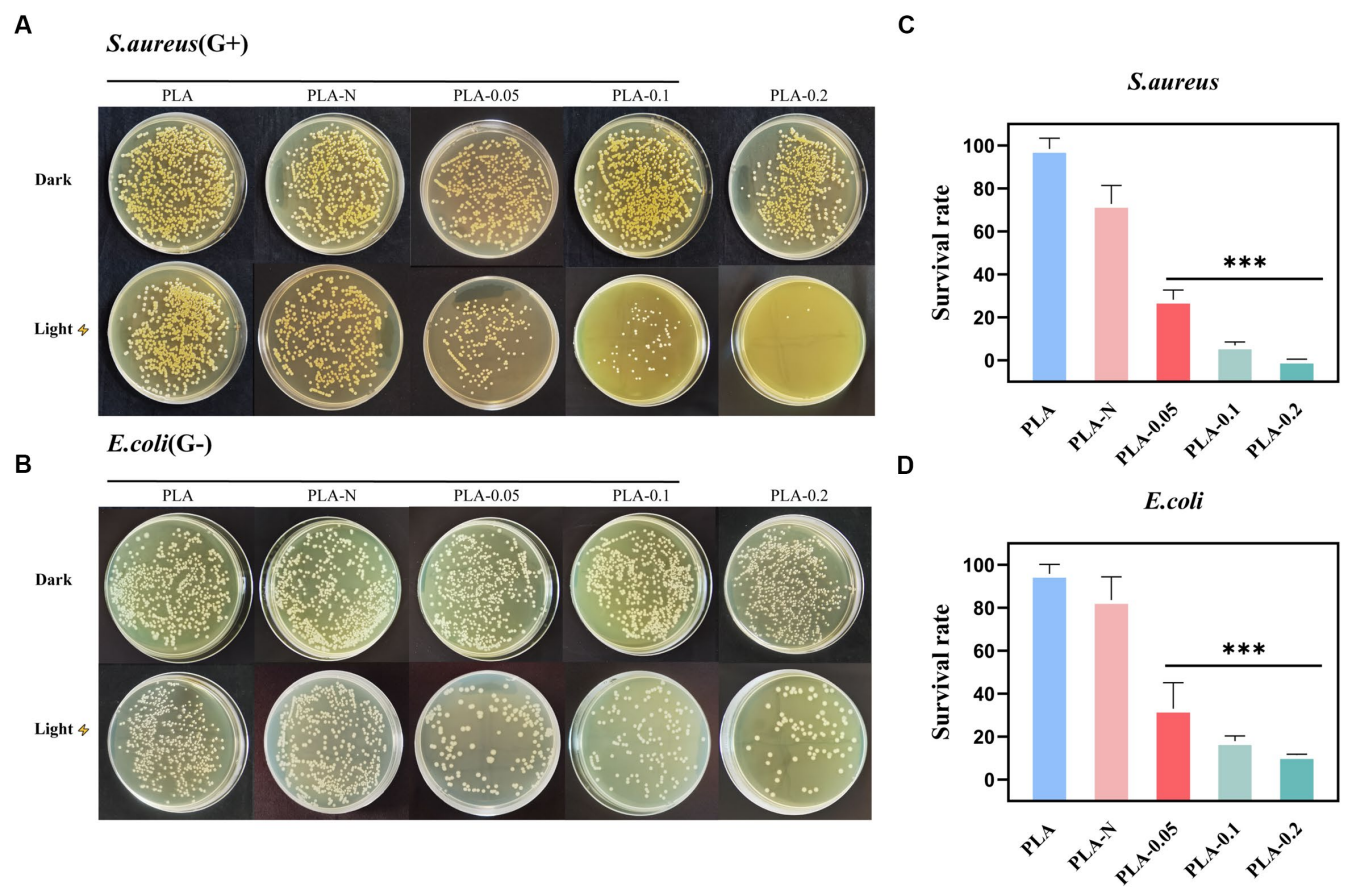
The antibacterial activity of various PLA membranes. **(A)** TSB-agar plates photographs of residual *S. aureus* on various PLA membranes under the different antibacterial treatments (dark or light irradiation for 30 min). **(B)** LB-agar plates photographs of residual *E. coli* on various PLA membranes with the different treatments (dark or light irradiation for 30 min). **(C,D)** Corresponding *S. aureus* and *E. coli* bacteria survival rate of various PLA membranes after irradiation treatments (*n* = 3, * represent significant differences. ***p < 0.001).

The antibacterial dressings containing photosensitizers capable of generating ROS upon irradiation were employed in antibacterial photodynamic therapy. ROS are molecules that gain electrons and are reduced to free radical forms, including superoxide anion O_2_^·−^, peroxide O_2_^·−^, hydrogen peroxide H_2_O_2_, hydroxyl radical OH, and hydroxyl OH− ions (Dryden, 2018). The primary mechanism by which ROS eliminates bacteria is through the disruption of bacterial cell walls and internal structure as well as the interference with normal physiological activities. In the typical photocatalytic antibacterial model, ROS can inflict structural damage on the bacterial membrane and induce perforation, thereby impairing its semi-permeability and resulting in the loss of bacterial contents. Furthermore, ROS can also induce damage to the DNA, mRNA, ribosomes, and essential proteins in bacteria (Pelgrift and Friedman, 2013; Thakur et al., 2019; Huo et al., 2021). Various unique mechanisms endow ROS with exceptional efficiency and superiority. This disruption leads to an imbalance in material exchange and ultimately leads to bacteria death. It is worth noting that metal oxides such as ZnO and TiO_2_ NPs also have a bactericidal effect after direct contact with bacteria under non-light conditions, which may be related to the positive charge on the surface of the nanoparticles (Zhang et al., 2021). In order to confirm the antibacterial efficacy of various PLA membranes, CFU reduction and absorbance curves were employed to assess their antimicrobial properties. *S. aureus* and *E. coli* were, respectively, employed as the representative strains of Gram-positive and Gram-negative bacteria to evaluate the antibacterial efficacy of PLA-N, PLA-0.05, PLA-0.1, and PLA-0.2. Pure PLA was set as the blank control. As shown in Figure 5, the inhibitory effect of PLA and PLA-0.05 on bacterial growth was found to be negligible. Furthermore, the antibacterial efficacy against Gram-positive (*S. aureus*) bacteria was more pronounced compared to that against Gram-negative (*E. coli*), especially in PLA-0.1 and PLA-0.2.

The survival rate changes of *S. aureus* are shown in Figures 5A,C. After 30 min of irradiation, all groups except PLA-N were effective in eliminating *S. aureus*. The *S. aureus* average survival rate of PLA-0.05, PLA-0.1, and PLA-0.2 was 28.29, 6.98, and 0.38%, respectively. However, as shown in Figures 5B,D, the survival rate changes of *E. coli* showed that the *E. coli* average survival rate of PLA-0.05, PLA-0.1, and PLA-0.2 was 33.07, 17.99, and 11.34%, respectively. With the increase in Ce-N co-doping ratio, the photocatalytic activity was enhanced, resulting in the increased production of ROS and consequently improved the antibacterial efficacy. Previous studies have indicated that photocatalytic or photodynamic antibacterial materials exhibit relatively weaker antibacterial efficacy against Gram-negative (G-) bacteria in comparison with Gram-positive (G+) bacteria (Sobotta et al., 2019; Songca and Adjei, 2022). Due to the unique structure of the cell wall in G- bacteria, it is more difficult for ROS to penetrate and exert their effects. This also explains the low efficiency of inhibition against *E. coli* in this section.

**Figure 6:**
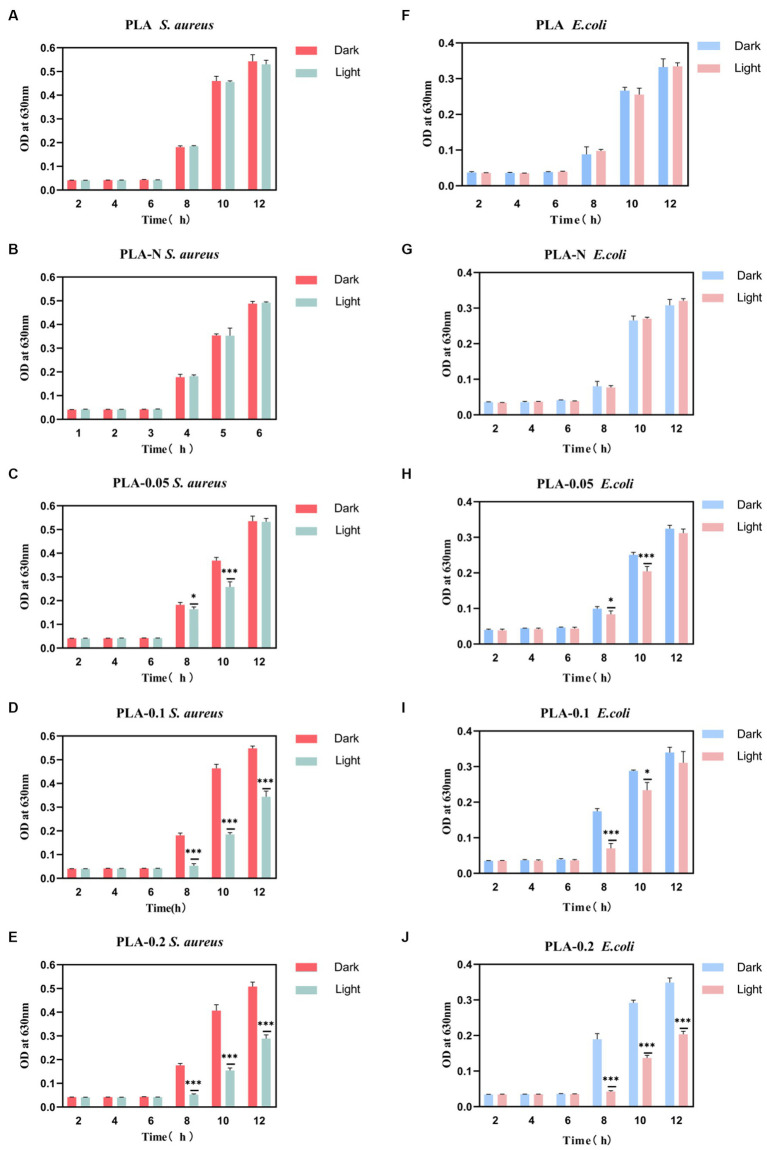
The absorbance trend at OD630 nm of *S. aureus* and *E. coli* bacterial suspensions under different treatment conditions (dark or light irradiation for 30 min) during a specific incubation time interval. **(A)** PLA *S. aureus*, **(B)** PLA-N *S. aureus*, **(C)** PLA-0.05 *S. aureus*, **(D)** PLA-0.1 *S. aureus*, **(E)** PLA-0.2 *S. aureus*, **(F)** PLA *E. coli*, **(G)** PLA-N *E. coli*, **(H)** PLA-0.05 *E. coli*, **(I)** PLA-0.1 *E. coli*, and **(J)** PLA-0.2 *E. coli* (*n* = 3, * represent significant differences. *p < 0.05, *** *p* < 0.001).

Figure 6 shows that PLA-N had minimal impact on the changes in optical density (OD) of the bacterial solution. However, as shown in Figure 6, the PLA-0.05, PLA-0.1, and PLA-0.2 groups exhibited growth inhibition during the logarithmic growth phase based on changes observed in OD values. In addition, the absorbance of *S. aureus* in PLA-0.05, PLA-0.1, and PLA-0.2 groups decreased significantly more than that of the *E. coli* group after irradiation.

### 3.6 Cytotoxicity assay

**Figure 7:**
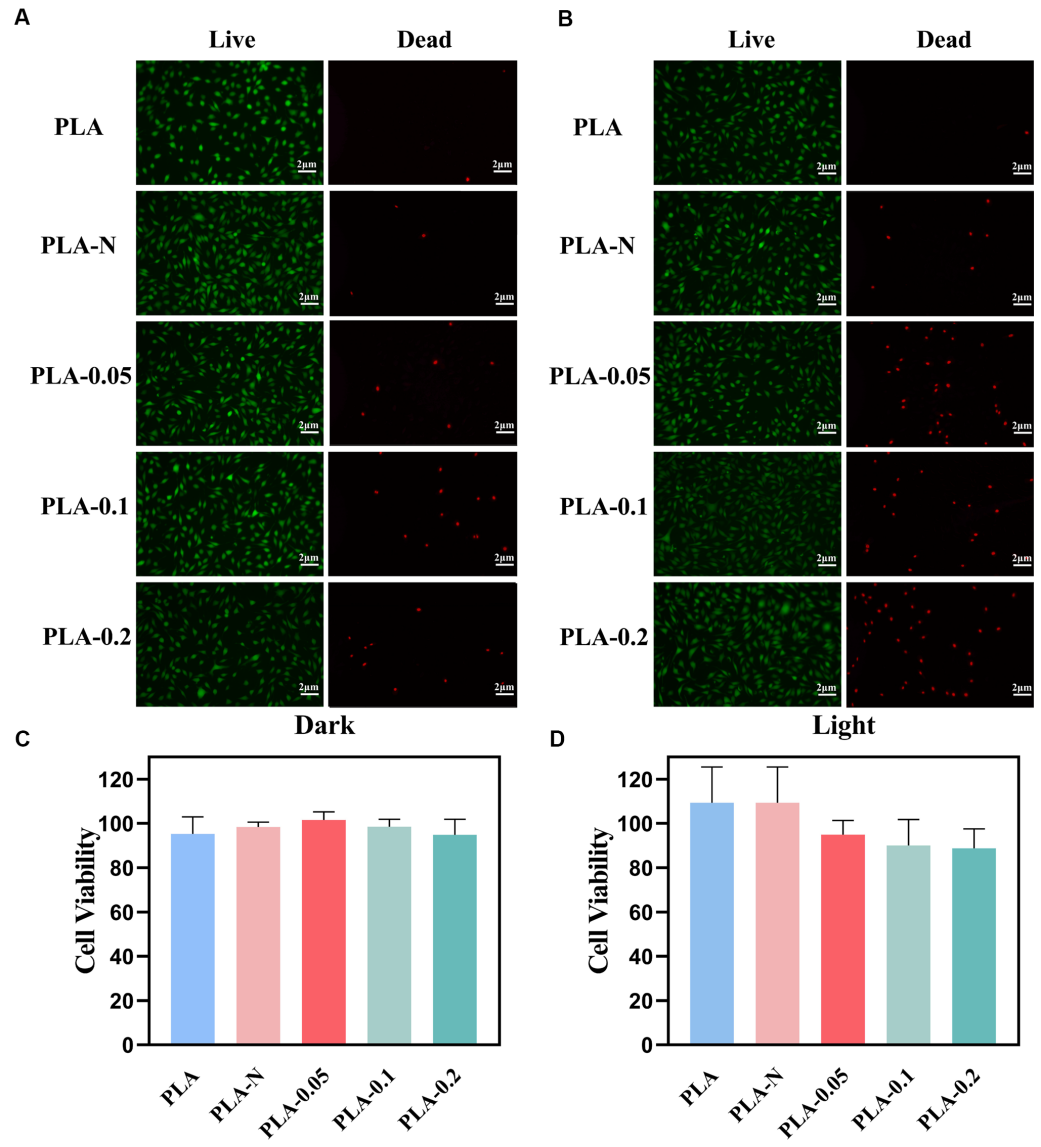
Biocompatibility evaluation of various PLA membranes. **(A)** Live/dead staining images of L929 cells under dark treatment. **(B)** Live/dead staining images of L929 cells under light treatment. **(C)** L929 cell viability under dark treatment. **(D)** L929 cell viability under light treatment (*n* = 3).

Fluorescence images of live and dead staining are shown in Figures 7A,B. Figures 7A,B represent experimental groups under dark and light conditions, respectively. As shown in Figure 7A, there were only a few dead cells in the PLA, PLA-N, PLA-0.05, PLA-0.1, and PLA-0.2 groups under the dark condition, which proved the good biocompatibility of the membranes under the dark condition. In Figure 7B, after irradiation for 30 min, there was no significant increase in the number of dead L929 cells in the PLA group compared with dark conditions. However, in PLA-0.05, PLA-0.1, and PLA-0.2 groups, the number of dead cells after light exposure was increased compared with that under dark conditions, especially for PLA-0.1 and PLA-0.2, which was consistent with the changes in cell survival rate by CCK8 assay.

The cytotoxicity of the as-prepared PLA membranes was evaluated by a cell counting kit-8 (CCK8) viability assay against the L929 cells line as shown in Figures 7C,D. After 24-h incubation at 37°C in the dark, all PLA, PLA-N, PLA-0.05, PLA-0.1, and PLA-0.2 mats exhibited cell viabilities exceeding 90%, indicating no significant cytotoxicity toward mammalian cells. Even after 30 min of irradiation, the cell viability remained above 80%. It is noteworthy that the cell viability of the PLA-0.2 group exhibited a relatively low level, which could be attributed to the impact of photocatalytic ROS generation on cellular (Holze et al., 2018).

## 4 Conclusion

In summary, N-element doped TiO_2_ and Ce, N elements co-doped TiO_2_ nanoparticles were fabricated by hydrothermal method. The doping of N and the co-doping of Ce and N could effectively enhance the phase of TiO_2_ transition from rutile to anatase, improve the crystallinity of the TiO_2_ NPs, and expand its utilization range of the visible spectrum, thereby enhancing photocatalytic activity. The PLA electrospun membrane loaded with 0.2Ce-N-TiO_2_ NPs exhibited excellent bactericidal performance against *S. aureus* and *E. coli* under visible light irradiation while maintaining relatively low cytotoxicity and excellent biocompatibility. Therefore, PLA-0.2 membrane, a polylactic acid polymer nanofilm equipped with photosensitizer for antibacterial activity through photocatalytic mode, holds great potential as a clinical antibacterial dressing in future.

## Data availability statement

The original contributions presented in the study are included in the article/supplementary material, further inquiries can be directed to the corresponding authors.

## Ethics statement

Ethical approval was not required for the studies on animals in accordance with the local legislation and institutional requirements because only commercially available established cell lines were used.

## Author contributions

HL: Data curation, Writing – original draft. XX: Methodology, Supervision, Writing – review & editing. SS: Investigation, Writing – review & editing. ZN: Investigation, Writing – review & editing. JL: Funding acquisition, Writing – review & editing. XL: Funding acquisition, Writing – review & editing.
