# Corrigendum: Polylactic acid electrospun membrane loaded with cerium nitrogen co-doped titanium dioxide for visible light-triggered antibacterial photocatalytic therapy

**DOI:** 10.3389/fmicb.2025.1587113

**Published:** 2025-04-16

**Authors:** Hanlin Lv, Xiaomin Xia, Sa Sun, Zhaojun Niu, Jie Liu, Xue Li

**Affiliations:** ^1^Department of Stomatology, The Affiliated Hospital of Qingdao University, Qingdao, China; ^2^School of Stomatology, Qingdao University, Qingdao, China

**Keywords:** photocatalytic therapy, antibacterial, electrospinning, Ce-N co-doped, polylactic acid

In the published article, there was an error in Figure 4, **page 9** as published. Due to an error that occurred inadvertently at the time of figure assembly, Figure 4I was incorrect.

The corrected Figure 4 and its caption appear below.

**Figure 4:**
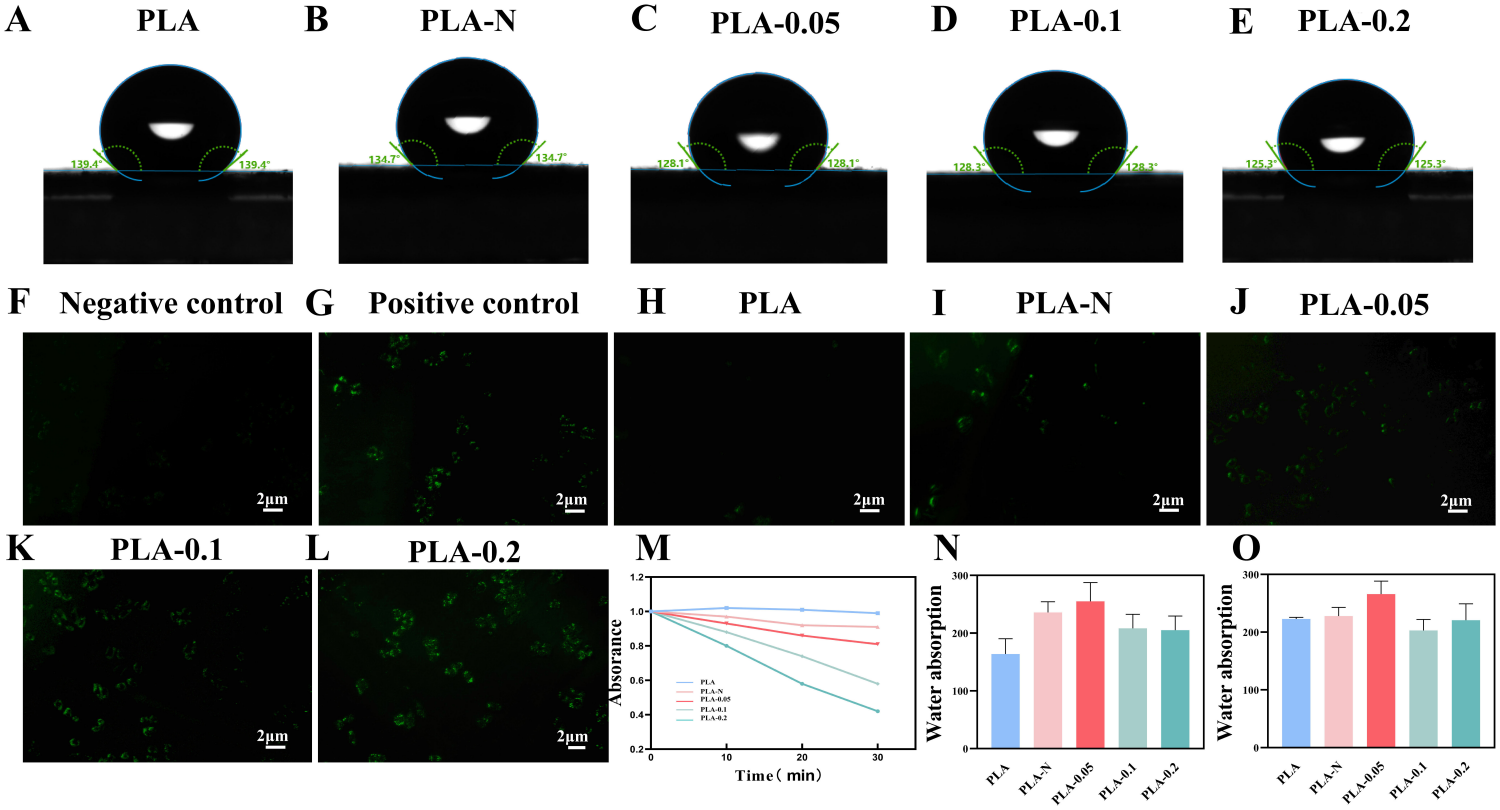
**(A–E)** Contact angle of PLA, PLA-N, PLA-0.05, PLA-0.1, and PLA-0.2 electrospinning membranes. Fluorescence microscope images of intracellular ROS generation induced by PLA, PLA-N, PLA-0.05, PLA-0.1, and PLA-0.2 with DCF-DA: **(F)** negative control, **(G)** positive control, **(H)** PLA, **(I)** PLA-N, **(J)** PLA- 0.05, **(K)** PLA-0.1, and **(L)** PLA-0.2. **(M)** Absorption curves of DPBF solution after incubation with different PLA, PLA-N, PLA-0.05, PLA-0.1, and PLA-0.2. **(N)** Swelling ratio of PLA, PLA-N, PLA-0.05, PLA-0.1, and PLA-0.2 electrospinning membranes at 2 h. **(O)** Swelling ratio of PLA, PLA-N, PLA-0.05, PLA-0.1, and PLA-0.2 electrospinning membranes at 24 h.

The authors apologize for this error and state that this does not change the scientific conclusions of the article in any way. The original article has been updated.

